# Comparison of Screws with Different Diameters in Subperiosteal Implant Application with Finite Element Analysis

**DOI:** 10.7150/ijms.93225

**Published:** 2024-10-07

**Authors:** Abdulsamet KUNDAKCIOGLU, Betul GEDIK

**Affiliations:** Istanbul University Faculty of Dentistry Department of Oral and Maxillofacial Surgery. Prof. Dr. Cavit Orhan Tutengil Street No:4 Vezneciler Fatih Istanbul Turkey.

**Keywords:** Finite Element Analysis, Dental Implantation, Subperiosteal Implant.

## Abstract

**Purpose:** This study aimed to assess subperiosteal implants concerning bone stress and screw displacement, utilizing finite element analysis to determine the optimal screw diameter for enhanced bone support.

**Methods:** Computed tomography data were translated into STL format, generating two skull models. Subperiosteal implants were constructed on these models and placed accordingly. Employing the finite element analysis method, screws with 1.5 mm and 2 mm diameter were inserted into one of the models to evaluate their impact under a 250 N chewing force.

**Results:** The 2 mm screw demonstrated superior performance compared to the 1.5 mm variant, showcasing reduced residual stress on the bone and implant. However, the 1.5 mm screw exhibited less implant movement.

**Conclusion:** The finite element analysis suggests the 2 mm screw diameter as more advantageous over the 1.5 mm variant for subperiosteal implants. Nevertheless, this investigation marks the initial stages in exploring this treatment option's potential.

## Introduction

Eliminating tooth deficiencies, restoring function and aesthetics are the leading issues in dentistry practice. One of the most frequently preferred methods to overcome these problems encountered worldwide is intraosseous implant treatments 1-4. Intraosseous implants developed by Brånemark need sufficient bone volume to be completely surrounded for proper function 5, 6. Therefore, a certain height and width of the alveolar bones are required for intraosseous implant treatment.

In patients who do not have satisfactory alveolar bone volume for implant placement due to various reasons, the chosen bone area need to be prepared before implant placement. Many grafting methods have been proposed in the literature to prepare the surgical area for implant surgery 1-3. Additionally, methods such as zygoma implants or angled implant placement techniques have been suggested in the literature to use the patient's residual bone 4. However, these treatment options also have their own complications and limitations 7. Prolongation of the treatment process due to the healing phase after grafting, creating an additional surgical area, sinus complications can be counted among these disadvantages 2, 3. In order to eliminate these restrictions and quickly restore the patient's function and aesthetics, personalized subperiosteal implants have been accepted as an increasingly used method in recent years 8-10.

The subperiosteal implant technique was first proposed in the literature in the 1940s. However, due to the limitations of production techniques, problems in bone implant compatibility and stabilization problems due to the lack of screw fixation, the use of such implants were extremely limited 11. Digital advances in computer-aided design and manufacturing software may reduce these problems and increase the indications of subperiosteal implants 12, 13. Moreover, as this type of implant can be applied without the need for additional surgery in Cawood IV, V and VI patients with severe bone resorption its popularity has increased in recent years 14. Subperiosteal implants enable elders with such extreme bone deficiencies to regain aesthetics and function more quickly 12, 15. Although its application requires high intra- and post-operative technical skills and knowledge, it is less traumatic for the patients compared to other alternatives 16-19.

Today, with the laser sintering method, which can be used clinically in the production of biomedical materials, it is possible to make personalized titanium implants that are highly compatible with the patient's bone under direction of tomography 20. Current subperiosteal implants can be fixed to the bone with mini screws in appropriate places of the jaws, and thus fixed prosthetic treatments can be performed in patients with severe bone deficiency without the need for extra grafting operations 21.

Another advantage in subperiosteal implant surgery is that there is no need to wait for the osteointegration process, unlike the intraosseous implant option. The long-term success of treatments using this technique depends on the immobility of the implants in the bone and hermetic closure by surrounding soft tissues. The most important elements to keep implant stability are three-dimensional (3D) implant design to receive ideal support from the bone surfaces during lateral forces and the bone resistance provided by the help of screws. To gain maximum strength with bone screws, appropriate diameter for screws should be prefered to provide optimum stability and durability. However, there are not enough studies in the literature regarding the ideal screw diameter that can withstand occlusal forces in subperiosteal implants. We aimed to determine the most suitable mini screw diameter by evaluating the behavior of mini screws of two different diameters (1.5 and 2 mm) under occlusal force using the finite element analysis method.

## Material and Methods

In our finite element analysis study, the bone model and its quality were critical components. Between 2018 and 2021, we evaluated 49 patients seeking implant treatment at our clinic who, based on clinical and radiographic examinations, were found to have insufficient bone tissue for conventional implants. Of these, 33 patients were unsuitable for custom subperiosteal implant treatment due to factors such as uncontrolled comorbidities, bisphosphonate use, cleft lip and palate history, or smoking. All patients were over 60 years old. Despite meeting the criteria, four patients opted out of the treatment. Subperiosteal implants were ultimately administered to 12 patients. For 11 of these patients, pre-operative and post-operative CT scans were captured as volumetric binary files (VBF) and grouped into a file cluster. Using the Model to Model Distance Module in 3DSlicer, we computed a distance map between the 11 models, generating point-to-point distance tables from anatomically selected points. A principal component analysis module then determined a mean value for the group, which was used to create a template model with the Shape Variation Analyzer module. The Shape Population module visualized this template, resulting in a 3D model that informed all subsequent subperiosteal implant designs. This generated model incorporated mean values from the 11 patients' data, ensuring all anatomically relevant points were included.

**Figure 1:**
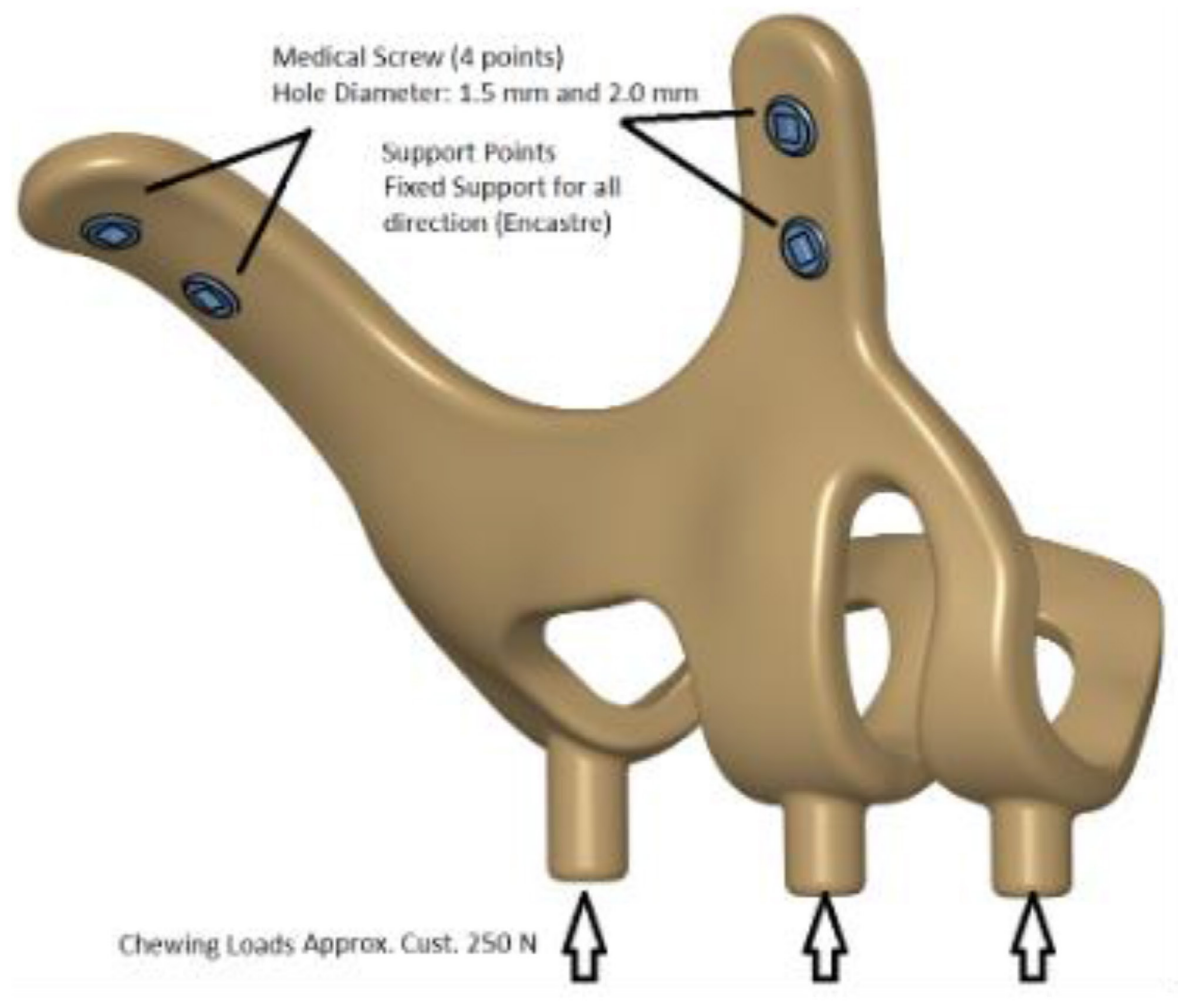
3D CAD geometry and boundary condition for implant models.

The maxillofacial models to be used (M1, M2) were provided by the medical design company BioTechnica (Turkey). The first step of the study was to convert these models into Stereolithography (STL) format. Later, computer-aided design (CAD) software was preferred to process the STL format. The reverse engineering module of the CAD software was used to convert the 3D models taken as point clouds into solid models. Thus, the 3D solid model necessary for the analysis of the implant geometry was obtained (Figure 1).

Since it is of great importance to minimize the amount of deviation between the resulting 3D model and point cloud data, deviation analysis was performed for all surfaces obtained with region definitions. As a result of the analysis, the deviation amount was determined as 0.05 mm.

**Figure 2:**
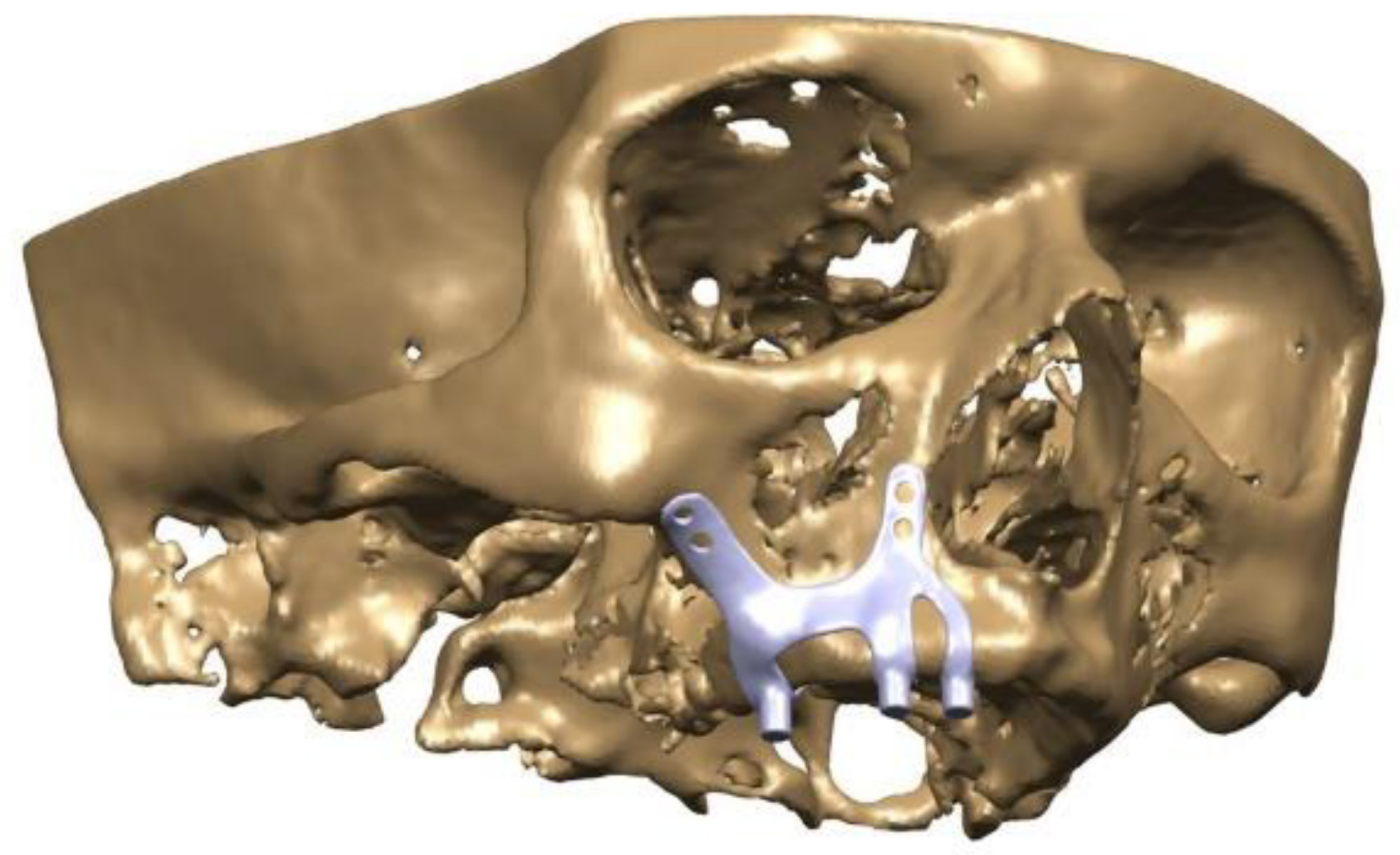
CAD model image of implant integrated on maxillary bone.

**Figure 3:**
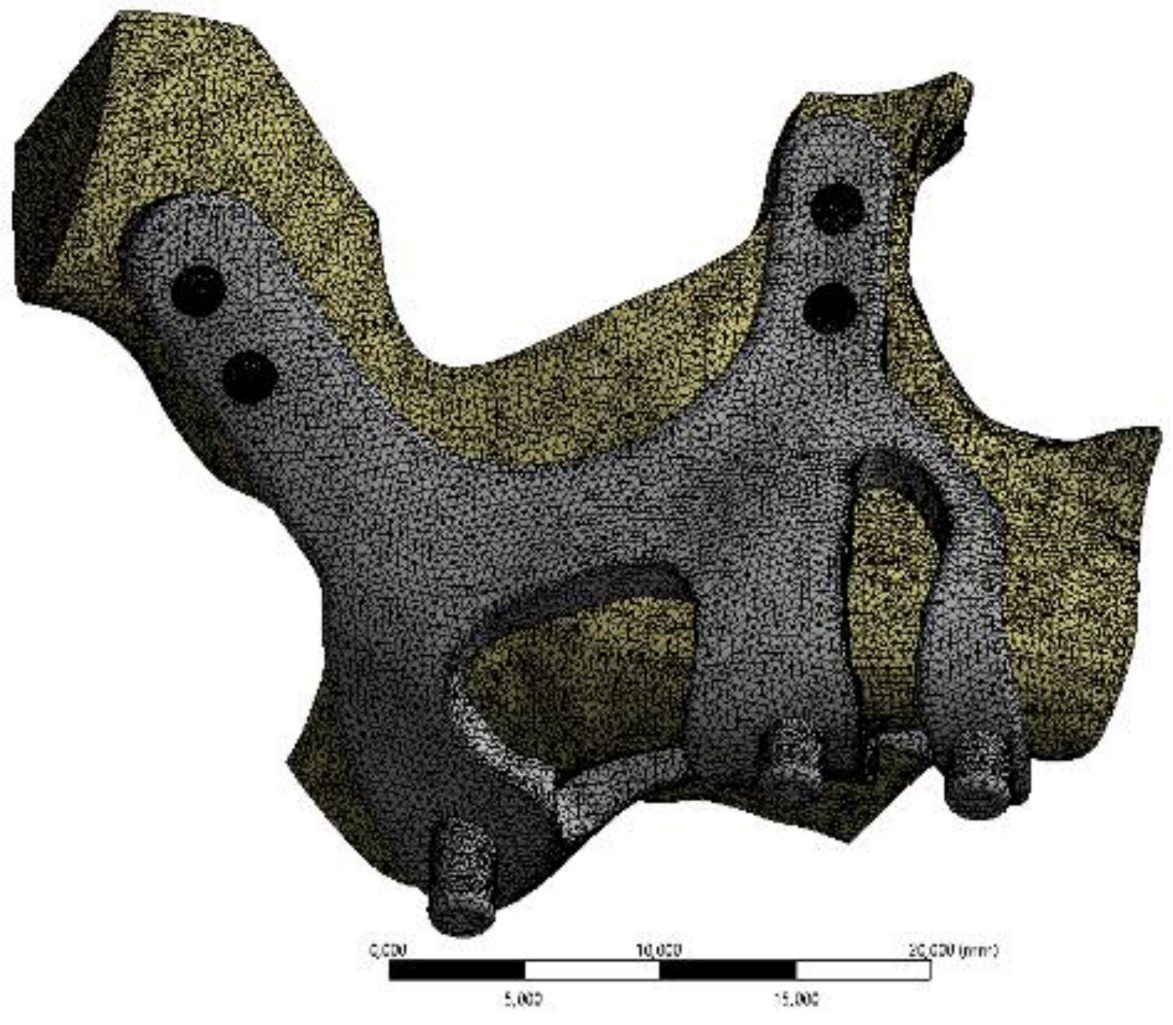
Image of implant and maxillary bone geometry mesh model.

Finite element analysis (FEA) software was used to determine the stress distribution over the entire unit consisting of bone and implants. The 3D solid model obtained with CAD was transferred to FEA and an adaptable 3D solution network was created in the finite element model with Mesh Generation. The solution matrix was calculated as a tetrahedron mesh type and a parabolic element. The mesh size was calculated as 0.5 mm (Figure 2, Figure 3). The mesh sizes used in the parts forming the whole, the elastic modulus and Poisson ratios of the materials used are given in Table 1 and Table 2.

In the continuation of the study, 250 N was applied vertically to the models. The stress distributions formed in two different models as a result of the application of force were examined. Data of maximum and minimum principal stresses (highest and lowest residual stress levels) in the bone were recorded. Additionally, stress distributions on the implants were evaluated with Von Mises data. In this way, the analyses made for intraosseous implants and the evaluations made with subperiosteal implants were handled separately. For comparability, the stress values of the implants were taken from the areas where intraosseous implants were applied.

## Results

**Figure 4:**
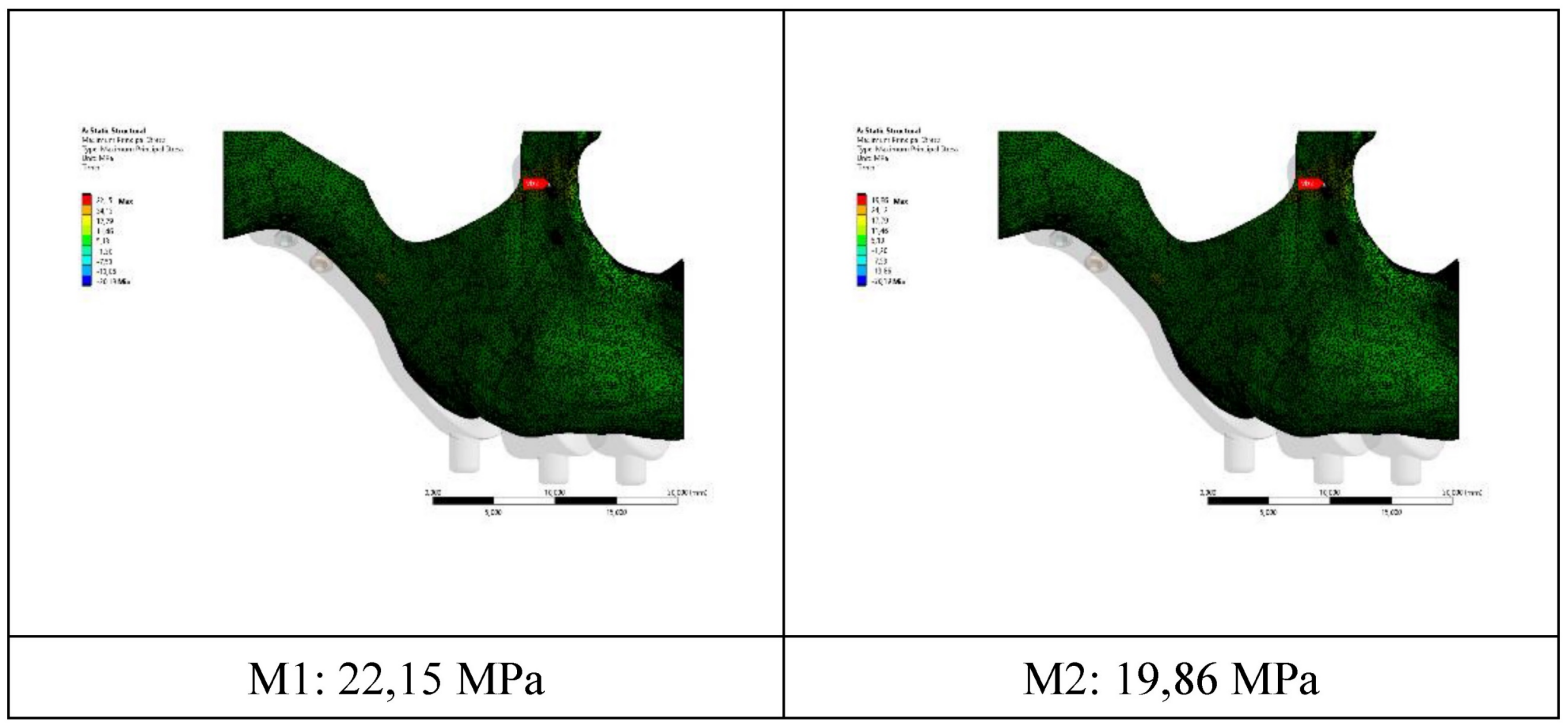
Changes in maximum residual stress on bone.

The residual stress values formed in the bone as a result of vertical loading were measured as 19.86 MPa in the M2 model with 2.0 mm diameter. The stress in the M1 model which has screws with 1.5 mm diameter was measured as 22.15 MPa. Increasing the diameter of the connection holes drilled into the bone enabled the stresses on the bone to be absorbed in a wider area. On the other hand, thinner implants had a negative effect on residual stresses due to the displacement force of the pressure applied to the bone. In addition, this effect caused an increase in the stress on the implant (Figure 4).

**Figure 5:**
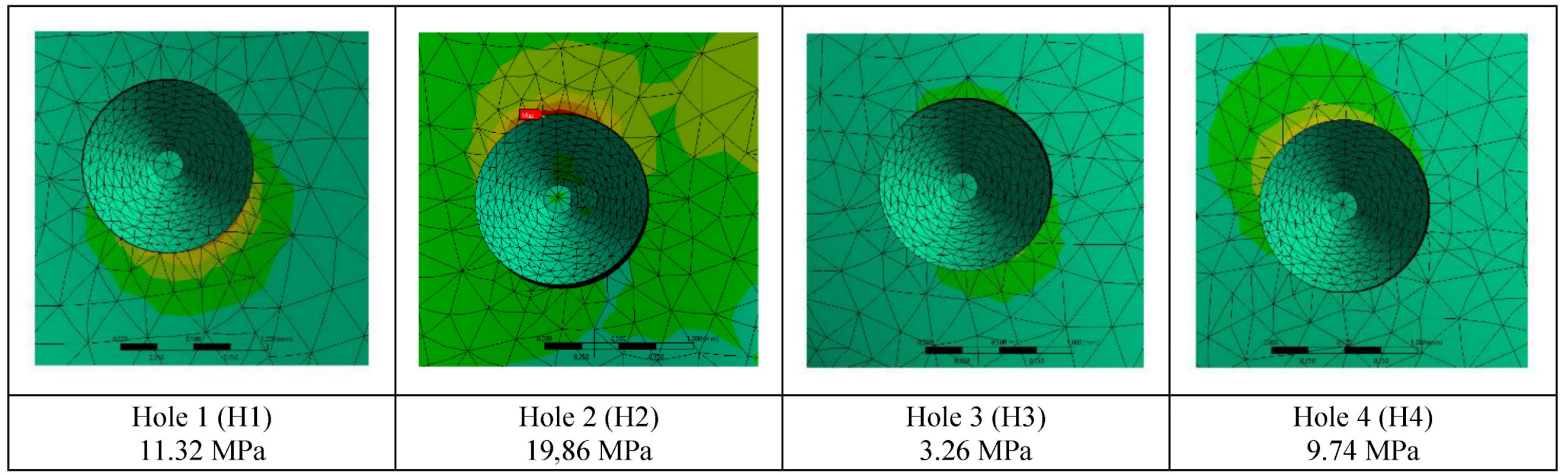
Changes in maximum residual stresses on bone with M2 configuration.

The stress on the bone decreases as the hole diameters increase, regardless of the implant thickness. Similar loads at the connection points were distributed over wider surfaces with wider diameter holes (Figure 5).

**Figure 6:**
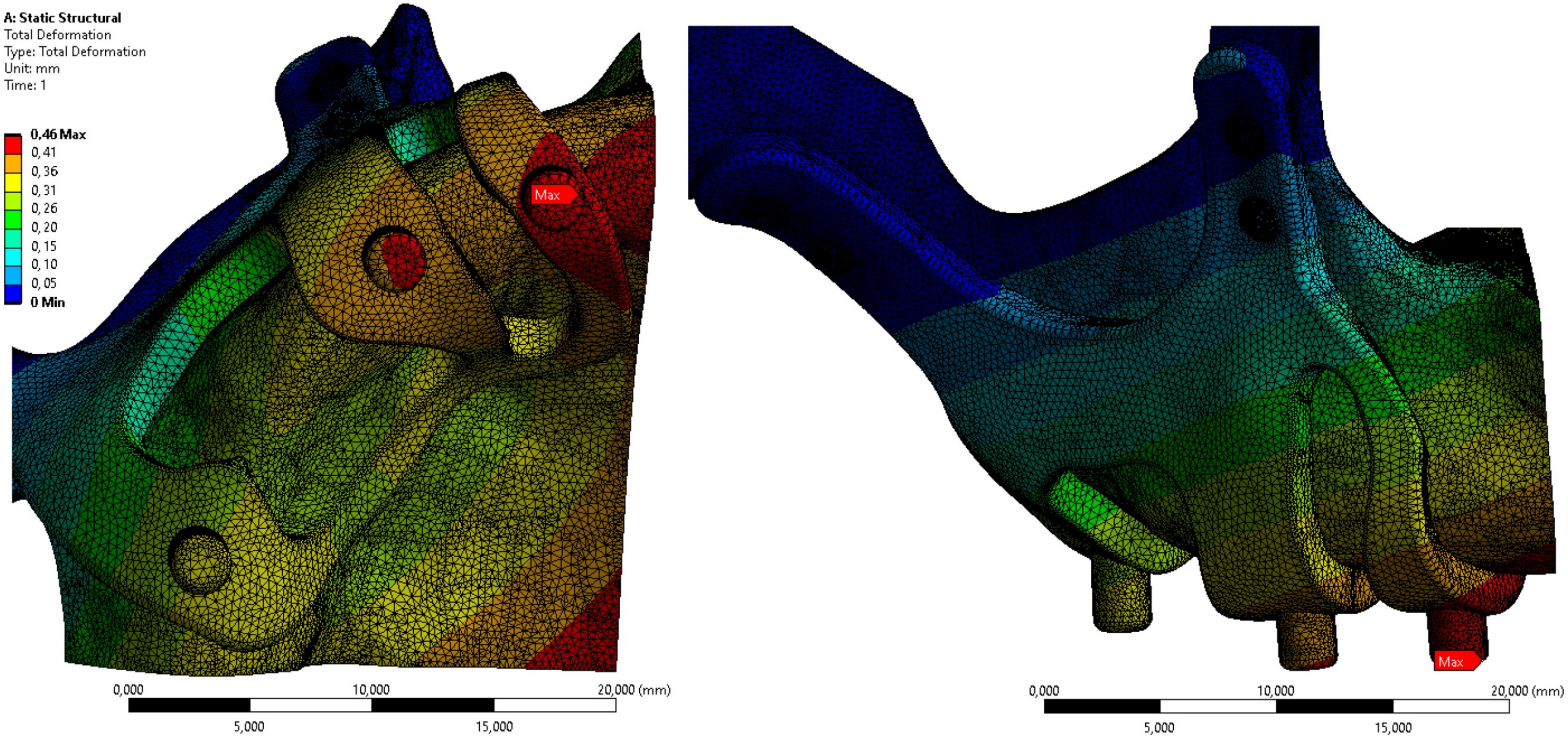
Displacement values on bone in M2 configuration.

Displacement value on the M2 model, which has the least residual stress on the bone, is given in Figure 6. When the axial and total displacement values were examined, the lowest displacement value on the implants was 0.42 mm in the M1 implant with 1.5 mm diameter and 2 mm thickness. In the M2 model configuration, a total displacement of 0.46 mm occurred in all directions (Figure 6).

**Figure 7:**
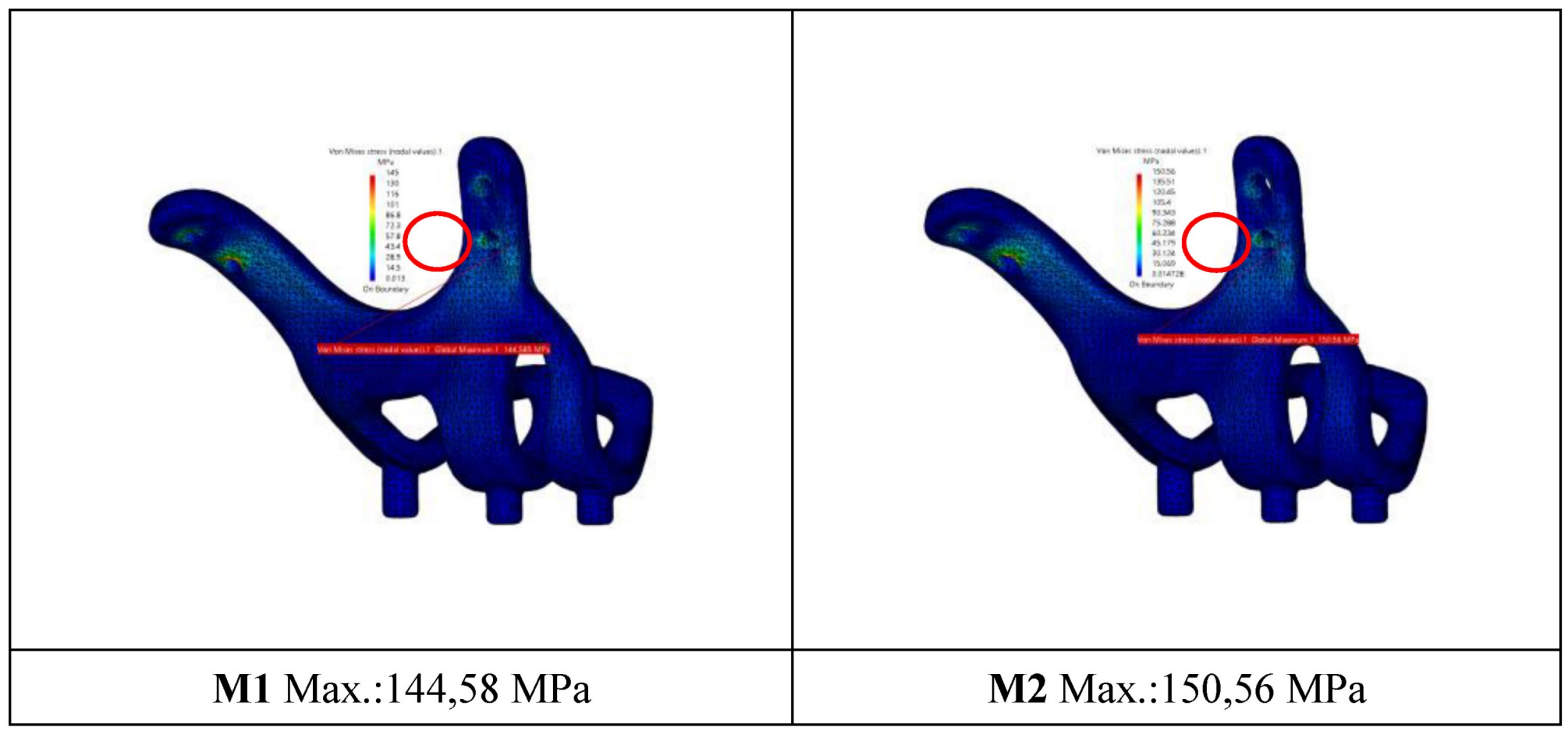
General view of von Mises stress values on the implants.

**Figure 8:**
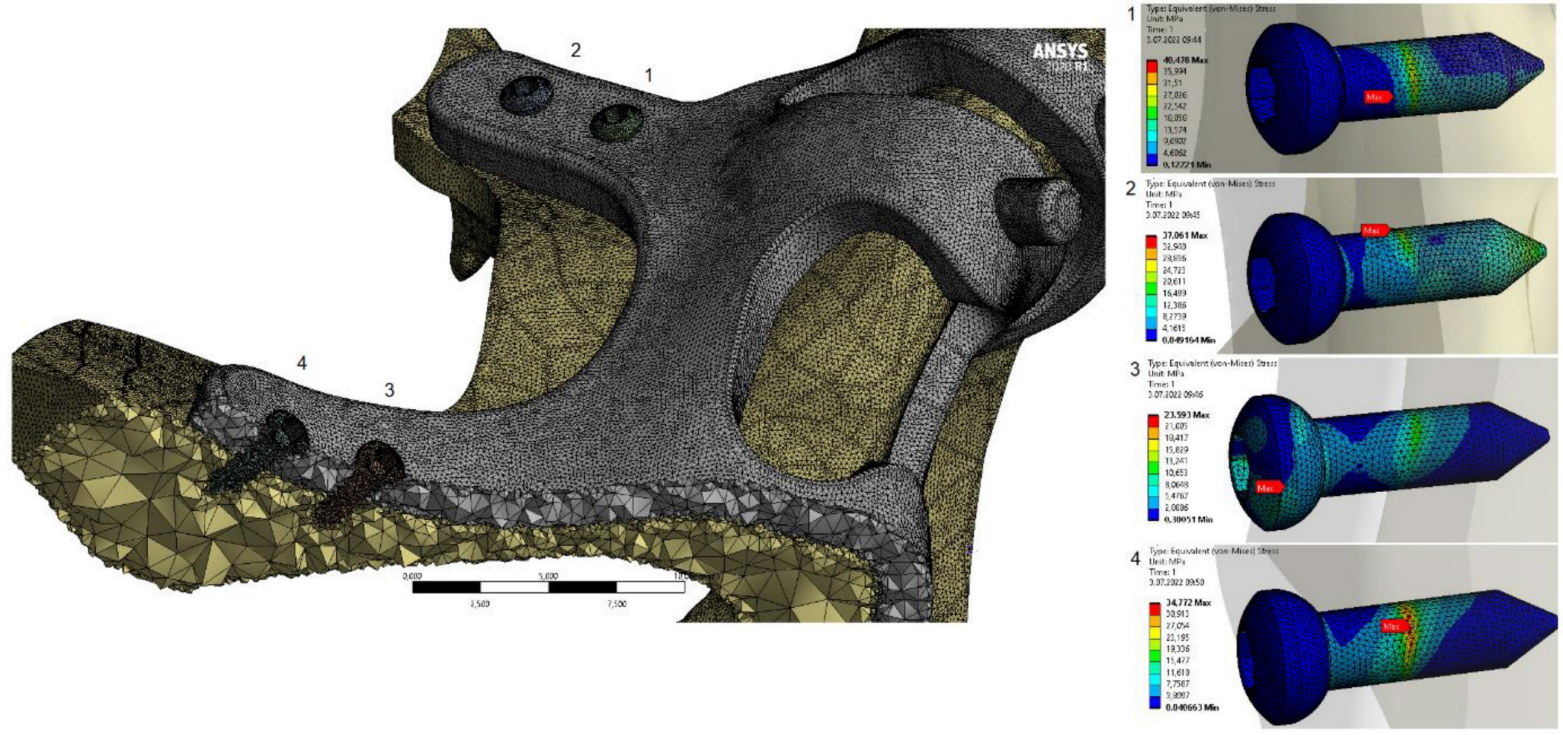
General view of von Mises stress values on connectors with M2 configuration.

When the von Mises stress levels in the implant were examined, the highest stresses were seen at the bolt connection interfaces in both models. It was determined that the highest stresses occurred in the M1 model with 2 mm thickness and 1.5 mm hole diameter. The stress accumulated in a high amount at thin-walled joint interfaces (Figure 7, Figure 8).

## Discussion

The subperiosteal implant concept is a one-stage surgery system applied to patients with excessive bone loss due to various reasons without the need for secondary procedures such as bone horizontal and vertical augmentation. The biggest advantage of this system is that it can be produced individually to minimize compatibility problems.

The first example of the subperiosteal implant concept in history can be seen in the studies of Dahl during the 1940s 11. This discovery was followed by the publication of the first case series by Goldberg in the United States 22. During these years, the size of the bone was measured by traditional methods for the construction of implants. After measurement subperiosteal implants were produced by casting in a laboratory environment. The surgical phase consisted of placing implants appropriate to the shape and size of the alveolar bone and covering these implants with the surrounding soft tissue. Post-operative stabilization was provided with the fibrous connection formed by the periosteum and the support of the neighbor regions. They were usually made of cobalt-chromium or titanium alloys, and the prosthesis was made using transmucosal abutments arising in the oral cavity. Due to the difficulties experienced in transferring the oral cavity to the model, laboratory problems in production and practice, its use could not spread to the general public so it was replaced by intraosseous implants 5.

With the development of technology and 3D software, in the mid-1980s, Dr. Carl Deckard and Dr. Laser proposed their sintering method and it was further developed by Joe Beaman. In the 3D laser sintering method, the desired product is obtained by selectively sintering a blocky material with a laser. With this method, production can also be made using many different materials. In this way, errors caused by measurement and laboratory processes can be prevented 23. In addition, virtual design can be corrected in this technique in case of need, it is possible to drill screw holes on the implant for proper fixation so that the implant receives maximum resistance from the bone. Adequate clinical case studies have been conducted about subperiosteal implant surgeries but none of them focused on mechanical and morphological properties of these implants 20, 24. One of the shortcomings in this area is the effect of screw diameters on stabilization which has not been studied on yet. Similar to our approach, various studies using finite element analysis (FEA) have explored the mechanical behavior of different dental materials and implants, providing critical insights into their stability and performance 25-27.

Plates and mini screws, which were first recommended by Michelet in 1973 for osteosynthesis in the jaws, have become the gold standard for the healing of maxillofacial fractures and osteotomies over the years 28. The use of screws of different thicknesses has been suggested for different surgeries as it varies according to the type of the surgery or the place where it is performed. While micro screws are used in intraoral autogenous grafting, mini-screws are used in cases of fracture or orthognathic surgery. In cases of mandibular or maxillary resection, 2.7 mm thick screws are used for reconstruction 29.

In our study, a comparison of 1.5 and 2 mm screws, which are frequently used in maxillary fractures and orthognathic surgery operations, was made. In clinical use, 1.5 mm screws are felt less by the patient than 2 mm screws as the 1.5 mm screws are fixed to the bone with a thinner-walled plate. Reconstruction screws were not included in the study as they would have caused bone traumas, especially to the very thin aperture piriform rim region.

Studies conducted over the years have shown that the support provided by screws and bone is leading point in terms of stabilization and resistance to occlusal forces. In this study, the effect of occlusal forces was investigated by fixing two subperiosteal implants with the same design with 2 different screw diameters. Screws with 1.5 and 2 mm diameters were chosen to compare the effect of screw diameter on subperiosteal implants. The aperture piriformis and the zygomatic buttress regions are the areas that remain unaffected by ongoing bone resorption. Because of that feature, they were preferred as the most ideal areas where we could place the mini-screws.

A force of 250 N was applied to the implants in our study. The residual and Von Mises forces on the implants under this force, displacement amounts and residual stresses on the bone were measured. The residual stresses in the M2 model, in which 2 mm thick screws were used, were found less than in the M1 model. The increase in the diameter of the holes drilled on the bone allowed the residual stress to spread over a wider area; therefore, the increase in the hole diameter caused a reduction in the residual stress value. This reduction is especially important in maintaining the integrity of the bone in thin bone areas such as the aperture priform area.

When the movements in the implant after the applied chewing force were measured, the amount of movement in the M2 model was 0.04 mm more in the region in which the most movement occurs compared to the M1 model. Considering these results, it was concluded that 2 mm screws were better than 1.5 mm screws in terms of both the stress they create on the bone and the formation of the implant, but the 1.5 mm screw showed better results in terms of stabilization of the implant.

Personalized subperiosteal implants have become an increasingly popular and promising topic in recent years. Publications concerning personalized subperiosteal implants are increasing in the literature, but in general, these publications focus on implant design and case follow-up 19, 30. There has been no study in the literature evaluating the effect of fixation screw diameters on the implant and residual bone.

Orthognathic surgery, maxillomandibular fracture, or orthodontic anchor screws are the most common studies on screws. In a study, the diameter and length of the mini-screws used for orthodontic anchorage were examined histomorphometrically. It was found that the increase in the diameter of the mini screws had a positive effect on the stability, but the length of the mini screws did not have a significant effect on the stability 31. A three-dimensional finite element model of the mandible was developed to simulate and examine the biomechanical loads of osteosynthesis screws in bilateral sagittal osteotomy. A 2.0 mm diameter mini screw was capable of providing sufficient stability at the osteotomy site after ramus split osteotomy. Even screws with a diameter of 1.5 mm were capable of standing forces of up to 89.5 N, a force normally unavailable to patients after ramus split osteotomy during the early recovery period. The forces applied by the patients after bilateral ramus split osteotomy did not exceed these values. The 2 mm screw diameter was capable of withstanding maximum chewing forces, whereas the 1.5 mm screw diameter could only withstand low chewing forces. It was concluded that the use of 2.0 mm bicortical titanium screws placed following sagittal split osteotomy provided sufficient stability in the osteotomy line 32.

Sindel *et al.*, in their study in 2014, examined the effect of bicortical screws of different thicknesses and numbers on stabilization after sagittal split osteotomy with the FEA method and concluded that no significant difference in stabilization between 1.5 and 2 mm screws existed 33. Molon *et al.* performed sagittal split osteotomy on *in vitro* models and compared 1.5 and 2 mm screws in terms of stabilization against occlusal forces, and they found no statistical difference between 1.5 and 2 mm screws 34.

Nagasao *et al.* examined 20 maxilla models of different thicknesses using the finite element analysis method in their study and concluded that the highest success in stabilization of the Le fort osteotomy was obtained when the screw diameter and bone thickness were the same 35. This result may explain the reason why the 1.5 mm screw diameter is more stable in the maxilla anterior region. Thin implant systems provide more stable results in areas where the bone is so atrophic.

For this reason, while custom-made implants are being produced, it can be planned with different screw diameters according to the bone thickness based on the tomography. Thick-diameter screws in the zygomatic buttress region and thin-diameter screws in the aperture piriformis region can be used in combination to achieve optimum results. The stabilization of the implant can be increased by providing additional supports with designs in which screws are placed in areas such as the palatal bone.

## Conclusion

Mini screw-supported personalized subperiosteal implants are a very new topic in oral surgery literature. However, early studies show promising results in severe bone resorption. The biggest advantage of this method compared to other options is that the patient receives the prosthesis immediately and does not need additional grafting surgery. Moreover, operation time is shorter in these surgeries and the interventions are less traumatic for the patients.

According to this finite element study, it was concluded that the 2 mm screw diameter is more useful than the 1.5 mm screw diameter. It was observed that as the diameter increases, the spread of the force over a wider area in the connection areas causes a reduction in the stress. However, the displacement value of the implant with a hole diameter of 2 mm was greater. As a result, the screws with 2 mm hole diameter caused less incoming and residual stress, but showed more displacement value than the 1.5 mm screws.

It seems that we are still at the beginning of the use of this treatment option. This method will evolve in the light of more research, development and long-term clinical follow-up. We recommend finite element analysis as a method for clinical applications but it should also be supported by following clinical studies.

## Figures and Tables

**Table 1:** Ti6Al4V mechanical feature.

Tensile Strength(MPa)	Modulus of Elasticity(GPa)	Yield Strength(MPa)	PoissonRatio	Density(kg/m^3^)	Hardness (Hv)
960-1270	100-120	830	0.33	4430	320-370

**Table 2:** Number of elements used in finite element solutions.

Model	Mass (gr)	Volume (mm^3^)	Mesh size (mm)	Number of Nodes	Number of Elements
**M1** - K:2.0mm, D:1.5mm	4.80	1076	0.5mm	142020	87879
**M2** - K:2.0mm, D:2.0mm	4.76	1068		141923	87843
